# Wavelength–angle characterization of high-energy X-ray beams from lapped Si(111) double-crystal monochromators using rocking-curve tomography

**DOI:** 10.1107/S1600577526004972

**Published:** 2026-06-16

**Authors:** Hiroshi Yamazaki, Kazuhiko Tahara, Yasuhiro Shimizu, Haruhiko Ohashi

**Affiliations:** a Japan Synchrotron Radiation Research Institute 1-1-1 Kouto Sayo Hyogo679-5198 Japan; b RIKEN SPring-8 Center, 1-1-1 Kouto, Sayo, Hyogo679-5148, Japan; Brazilian Synchrotron Light Laboratory, Brazil

**Keywords:** lapped Si(111) double-crystal monochromator, wavelength–angle distribution of X-ray beam, full-rotation θ–2θ rocking-curve measurement, computed tomography, rocking-curve tomography, high-energy X-rays, lapped crystals

## Abstract

Lapping the surfaces of Si(111) crystals in a double-crystal monochromator (DCM) has recently been shown to increase the 100 keV X-ray beam intensity by nearly an order of magnitude, raising the need for a reliable experimental evaluation of the resulting beam quality, particularly wavelength spread (energy bandwidth) and angular divergence. To address this need, a method for determining the wavelength–angle distribution of high-energy X-ray beams is developed by interpreting rocking-curve measurements as projections in wavelength–angle (DuMond) space and reconstructing the distribution using an iterative computed-tomography approach. When applied to beams produced with polished and lapped Si(111) DCMs, the method reproduces the DuMond prediction for perfect crystals and reveals systematic changes in wavelength spread while the angular divergence remains nearly unchanged. These results demonstrate a practical framework for experimentally characterizing the wavelength–angle distributions of high-energy X-ray beams, providing a basis for understanding and optimizing beam properties beyond conventional one-dimensional metrics.

## Abstract 2

A computed-tomography method using full-rotation rocking curves is developed to characterize 100 keV X-ray beams from lapped Si(111) crystals, revealing systematic wavelength broadening while maintaining constant angular divergence in DuMond space.

## 1. Introduction

Accurate characterization of the wavelength–angle distribution of synchrotron radiation beams is essential for understanding and optimizing X-ray optical systems. Although rocking-curve measurements are widely used to characterize X-ray beams, they usually provide only limited one-dimensional information such as angular width or peak position. A method capable of experimentally determining the wavelength–angle distribution provides a more complete description of beam properties.

Lapping the surfaces of Si(111) crystals in a double-crystal monochromator (DCM) has been shown to increase the beam flux by factors of 7–11 in the high-photon-energy region around 100 keV (Yamazaki *et al.*, 2026 ▸).

Such a substantial intensity enhancement is highly attractive for a wide range of experiments; however, the wavelength spread (energy bandwidth) and angular divergence of the resulting beams—key parameters that determine their applicability as X-ray probes—have not yet been quantitatively characterized.

For beams produced by perfect-crystal DCMs, these characteristics can be fully predicted within the framework of the DuMond analysis (DuMond, 1937 ▸), which represents the beam in a two-dimensional wavelength–angle space (hereafter referred to as DuMond space). In this framework, Bragg’s law exhibits an intrinsic coupling between wavelength and reflection angle, while the finite angular acceptance of the crystal (the Darwin width) determines the effective filtering property. However, this prediction is no longer applicable to surface-lapped crystals.

A possible route to experimentally characterize wavelength–angle distributions of realistic beams is provided by the DuMond analysis itself. Consider that an analyzer crystal is placed in the path of the beam to be characterized. Rotating the crystal corresponds to shifting its wavelength–angle filter in DuMond space. Reflection intensity is given as the common area between the filter and the wavelength–angle distribution of the beam. Thus, a rocking-curve profile includes a specific aspect of the wavelength–angle distribution. Different aspects can be collected from the rocking curves for different crystallographic planes. Then, an inverse problem arises to estimate the original wavelength–angle distribution from such aspects. On this basis, Yamazaki & Ishikawa (2010 ▸) demonstrated that the wavelength–angle distribution could be reconstructed using a computed-tomography (CT) approach.

In the present study, this CT-based approach is further developed to enable experimental reconstruction of the wavelength–angle distributions of high-energy X-ray beams. By interpreting rocking-curve profiles as projections of the wavelength–angle distribution in DuMond space, the method provides a practical framework for characterizing the wavelength–angle distribution. As a representative application, the approach is applied to 100 keV X-ray beams produced using lapped Si(111) DCMs.

## 2. Rocking-curve tomography

This section describes the experimental and analytical framework used to determine the wavelength–angle distribution of high-energy X-ray beams. First, the physical relationship between a measured rocking-curve profile and the wavelength–angle distribution of the incident beam is formulated in terms of a Radon transform (Radon, 1917 ▸; §2.1). Practical constraints arising from the analyzer crystal, high photon energy, and measurement errors are then addressed (§2.2 and §2.3). Finally, an iterative reconstruction algorithm suitable for sparse and non-negative projections is described (§2.4).

### 2.1. Radon-transform expression of rocking-curve profile

**Figure 1:**
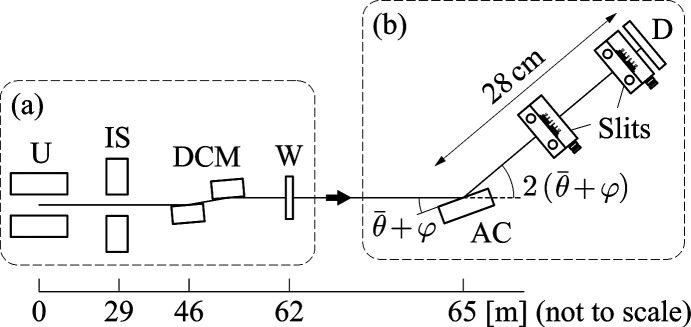
Optical layouts (*a*) to prepare the beams to be characterized and (*b*) to perform the rocking-curve tomography. Distances from the undulator (U) are indicated in metres. IS: incident slit; DCM: double-crystal monochromator; W: vacuum window; AC: analyzer crystal; D: detector.

Fig. 1 ▸ shows the whole experimental set up in this study. Part (*a*) is the optical configuration to prepare the X-ray beams to be characterized (§3), and part (*b*) is the apparatus for the rocking-curve tomography.

An analyzer crystal with lattice spacing *d* is placed in the path of the X-ray beam to be characterized. A reference wavelength 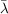 is chosen close to the mean wavelength of the incident beam, although it need not coincide exactly with it. The corresponding reference Bragg angle 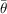 is defined by Bragg’s law, 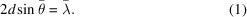
To obtain a rocking curve, the analyzer crystal is rotated by an angle φ about 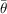. At each rotation angle, a fraction of the incident beam satisfying the Bragg condition is reflected and detected with a detector in the 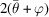 direction.

**Figure 2:**
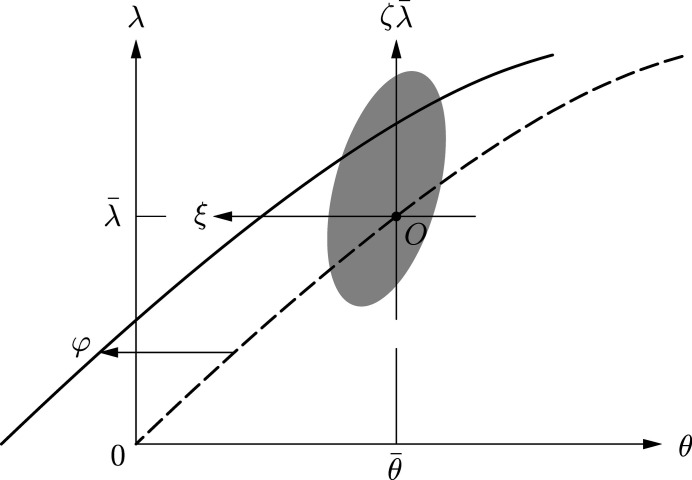
Schematic illustration of X-ray reflection in wavelength–angle (DuMond) space.

The reflection process can be conveniently described in the wavelength–angle space introduced by DuMond (1937 ▸), where the vertical and horizontal axes represent the wavelength λ and the angular deviation θ, respectively (Fig. 2 ▸). In this representation, Bragg’s law forms a curve that acts as a wavelength–angle filter. The incident beam occupies a finite region around the reference point defined by 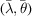. Rotating the analyzer crystal by φ shifts the Bragg-condition curve horizontally in this space, and the reflected intensity is given by the overlap between the shifted filter and the wavelength–angle distribution of the incident beam.

To formulate this relationship quantitatively, a local coordinate system is introduced with its origin at the reference point. The relative wavelength deviation is defined as 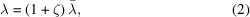
where ζ is dimensionless. The angular deviation is expressed by ξ, defined such that positive ξ corresponds to the direction of increasing crystal rotation angle φ.

**Figure 3:**
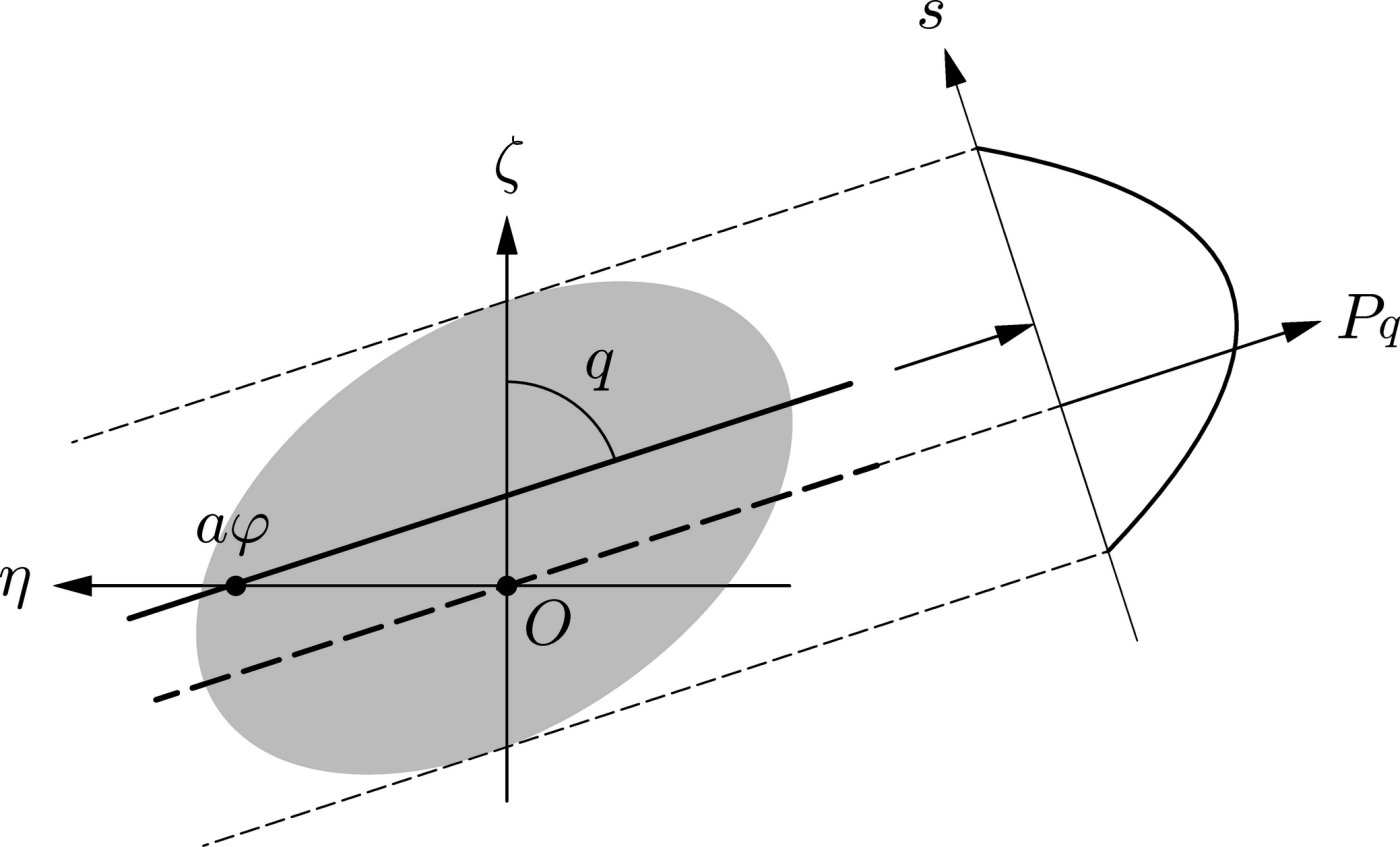
Magnified view of Fig. 2 ▸ around the reference point, shown in the scaled (ζ, η) coordinate system. The angular coordinate is rescaled by the aspect factor *a* to adjust the relative scaling between wavelength and angle.

For convenience in later analysis, a scaled angular coordinate η is further introduced as 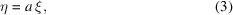
where *a* is a dimensionless aspect factor used to adjust the relative scaling between wavelength and angle (§2.2.3). Fig. 3 ▸ shows a magnified view of DuMond space around the reference point, expressed in the (ζ, η) coordinates. The introduction of the aspect factor modifies the apparent slope of the Bragg-condition line, facilitating a more uniform distribution of projection angles for high-energy X-ray beams.

When being able to be linearized within the range of the wavelength–angle distribution, Bragg’s law reduces to 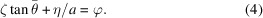
Let *D*(ζ, η) denote the wavelength–angle distribution of the incident beam, and it is intrinsically a non-negative function. The reflected intensity measured at a crystal rotation angle φ is then proportional to the integral of this distribution along the line defined by equation (4), 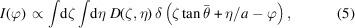
where δ is Dirac’s delta function.

Introducing a projection angle *q* defined by 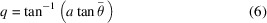
and a scaled coordinate 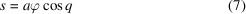
transforms the argument of the delta function into a standard Radon-transform form. Equation (5) can then be rewritten as 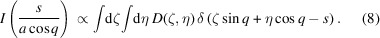
The right-hand side of equation (8) represents the Radon transform of the distribution *D*(ζ, η) at projection angle *q* (Kak & Slaney, 1988 ▸). Thus, a rocking-curve profile corresponds to a projection of the wavelength–angle distribution in DuMond space.

Denoting this projection by *P*_*q*_(*s*), and normalizing the measured rocking curve 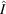 such that its integral over *s* is unity, the projection can be expressed as 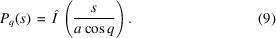
This formulation provides the basis for reconstructing the wavelength–angle distribution of the incident beam from a set of rocking curves obtained at different Bragg angles.

### 2.2. Technical details

This subsection addresses practical constraints and experimental considerations required for the formulation in §2.1 to remain valid. The selection of the analyzer crystal, conditions under which deconvolution can be avoided, and limitations specific to high-energy X-ray beams are discussed.

#### 2.2.1. Selection of analyzer crystal

Highly imperfect crystals cannot be used as analyzer crystals because spatially nonuniform lattice distortions disturb the Bragg condition in an uncontrolled manner. The use of highly perfect crystals is therefore essential.

Silicon is preferable to diamond because its larger lattice constant allows a greater number of crystallographic reflections to be accessed at a given wavelength. Rocking curves are obtained by performing θ–2θ scans with a Si(111) analyzer crystal finished by mechanochemical polishing. Its fundamental reflection provides the smallest Bragg angle, while higher-order *nnn* reflections are obtained in the upward-reflection geometry by further crystal rotation. Reflections from the corresponding negative planes 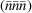 are also accessible in the downward-reflection geometry.

In the downward-reflection geometry, the glancing angle formally exceeds 90°. For convenience, angles larger than 90° are redefined by subtracting 180°; for example, a glancing angle of 170° is also represented as −10°.

Owing to the discrete crystallographic structure of the analyzer crystal, the available Bragg angles—and hence the projection angles in the Radon-transform representation—are discrete and sparse. This constraint directly influences the choice of reconstruction algorithm, as discussed in §2.4.

#### 2.2.2. Avoidance of ill-posed deconvolution

An intrinsic reflection profile defined by the analyzer crystal, which has a finite angular width (Darwin width), can be calculated using the dynamical diffraction theory. A measured rocking-curve profile for a realistic incident beam is represented as the convolution of the corresponding projection profile with this intrinsic profile. However, numerical deconvolution is a classic ill-posed problem (Tikhonov & Arsenin, 1977 ▸); it significantly amplifies high-frequency noise, often leading to numerical artifacts.

To ensure data integrity, the simplest strategy is to avoid deconvolution altogether. Assuming a Gaussian approximation for the sake of simplicity, a measured width five times larger than the intrinsic Darwin width corresponds to a broadening effect of approximately 2%. While the actual Darwin–Prins profile has longer tails than a Gaussian, this criterion effectively ensures that the physical properties of the incident beam dominate the measured profile, thereby preserving the integrity of the projection data without the need for unstable numerical deconvolution. If this condition is not met, the rocking curve is excluded from the reconstruction dataset.

In practice, significant contamination from intrinsic broadening occurs only when the beam from an *nnn*-reflection perfect-crystal DCM impinges on the analyzer crystal in the same *nnn* reflection—specifically, in the perfect-crystal non-dispersive (+, −, +) configuration.

#### 2.2.3. Avoidance of limited-angle problem for high-energy X-ray beams

This subsection addresses issues specific to high-energy X-ray beams and differs fundamentally from earlier implementations developed for moderate photon energies (Yamazaki & Ishikawa, 2010 ▸).

As the absolute value of the reflection order *n* of the analyzer crystal increases, the reflected intensity generally decreases and becomes increasingly susceptible to noise. In the present study targeting 100 keV X-ray beams, reflections up to |*n*| = 9 still provided sufficient signal-to-noise ratios. However, the corresponding Bragg angle for the 999 reflection is only approximately 10°. If the aspect factor is set to *a* = 1, the accessible projection angles are confined to within ±10°, and information near orthogonal projections (90°) is almost entirely lost. As a result, the reconstructed distribution suffers from severe limited-angle artifacts.

The introduction of *a* serves two primary objectives that are essential for rocking-curve tomography at high energies. First, it rescales the DuMond space such that the wavelength–angle distribution, which is originally highly elongated, becomes more isotropic. This adjustment facilitates more stable and balanced numerical processing during the iterative reconstruction. Second, it expands the range of effective projection angles *q*, which is a physical necessity for reliable image reconstruction.

In this study, *a* = 20 was chosen as a practical optimum to ensure sufficient angular diversity. With *a* = 20, the reflections from *n* = 1 to 7 are distributed over *q* ≃ 22° to 70°. While higher-order reflections such as *n* = 8 and 9 were available, they were excluded from the analysis because the concentration of their projection angles at large *a* would lead to another angular bias. This strategic selection effectively transforms the limited-angle problem into a well conditioned tomographic task.

### 2.3. Measurement errors and removal procedures

This subsection describes experimental imperfections that affect the measured rocking curves and the procedures used to mitigate their influence prior to reconstruction. The dominant contributions arise from stray scatter and detector offsets, electronic noise, and uncertainties in the crystal rotation angle.

#### 2.3.1. Stray scatter and detector dark current

To suppress stray scatter reaching the detector, two slits were installed on the detector arm, as shown in Fig. 1 ▸(*b*). Although this configuration significantly reduced background contributions, a residual baseline remained, varying with the detector rotation angle.

In addition, the peak intensities of different reflections spanned more than three orders of magnitude. Consequently, the amplifier gain had to be adjusted for each reflection, leading to changes in the detector dark current. These effects resulted in discontinuous baseline offsets in the recorded intensity profiles.

All such baseline contributions were removed by baseline correction prior to the CT reconstruction.

#### 2.3.2. Electronic noise

Detector signals inevitably fluctuate owing to electronic noise. After baseline correction, this noise can lead to small negative values in the rocking-curve profiles, which violate the non-negativity stemming from the non-negative wavelength–angle distribution.

This effect is particularly pronounced for weak reflections, where higher amplifier gains are necessary and the noise amplitude correspondingly increases. Numerical smoothing filters, such as the Savitzky–Golay filter (Savitzky & Golay, 1964 ▸), can effectively reduce these fluctuations, provided that they do not introduce peak distortion or Gibbs-type oscillations.

Residual negative values are further suppressed by adding a small positive regularization constant to the distribution during reconstruction (§2.4.2).

#### 2.3.3. Rotation angle of the crystal

The rotation axis of the analyzer crystal should be perpendicular to the incident beam to ensure that the measured rotation angles are precise. Although high-precision alignment methods for wavelength determination, such as Bond’s method (Bond, 1960 ▸), have been established, they are unnecessarily sophisticated for the present purpose.

Practically, minor misalignment can be tolerated and its elimination can be done in the numerical analysis. Such misalignment slightly affects the angular profile and central position of the measured rocking curve. In DuMond space, this can be interpreted either as a modification of the reference Bragg angle or a lateral shift of the measured rocking curve, within the linear approximation.

Such angular errors are corrected by laterally shifting the projection profiles during reconstruction. For beams from lapped-crystal DCMs, this correction had a negligible effect owing to their relatively broad reflection profiles. For the beam from the polished-crystal DCM, however, the correction was beneficial because of the much narrower profiles.

### 2.4. OS-EM reconstruction

Based on the formulation in §2.1, the determination of the wavelength–angle distribution from rocking-curve measurements is formulated as a tomographic reconstruction problem. After applying baseline corrections (§2.3.1) and numerical smoothing filters (§2.3.2) to the noisy measured rocking curves, the problem is characterized by the following conditions: (i) the wavelength–angle distribution is intrinsically non-negative; (ii) the projection angles are discrete and sparse; (iii) the projections may include lateral shifts that must be corrected (§2.3.3); (iv) the angular coverage can be made approximately uniform by adjusting the aspect factor; and (v) nevertheless, small negative values remain in the projection profiles.

Given conditions (i) and (ii), the reconstruction is naturally formulated within the framework of likelihood maximization using the expectation-maximization (EM) algorithm (Shepp & Vardi, 1982 ▸) and its variants. In this study, an extreme form of the ordered-subset expectation-maximization (OS-EM) algorithm (Hudson & Larkin, 1994 ▸) is adopted, in which each ordered subset consists of a single projection. This choice results in a row-action (sequential-update) EM algorithm and allows the lateral shift correction described in (iii) to be incorporated in the simplest manner. The approximate uniformity of the projection angles in (iv) further supports the use of subset partitioning. Residual negative values described in (v) are addressed through regularization.

Although the number of available projections is much smaller than in typical medical imaging applications such as PET or SPECT, this sparsity does not pose a serious limitation in the present case. The wavelength–angle distributions of interest are expected to be relatively simple; otherwise, the beams would not be suitable as general-purpose X-ray probes. Stable and physically plausible solutions can therefore be obtained with a limited number of projections.

#### 2.4.1. Outline of the reconstruction procedure

For clarity, the reconstruction procedure is outlined as follows.

The wavelength–angle distribution is first initialized on a discrete grid (§2.4.2). The reconstruction then proceeds through repeated OS-EM cycles, each of which consists of sequential updates using all available projection angles.

A single update for a given projection angle *q* comprises the following steps: (i) rotation of the current estimate by the projection angle *q*; (ii) forward projection of the rotated distribution; (iii) correction of the lateral shift of the measured projection; and (iv) backprojection update.

One OS-EM cycle is completed after the above update has been applied once to each projection angle in the prescribed order. The OS-EM cycle is repeated iteratively until convergence of the reconstructed distribution is achieved.

#### 2.4.2. Initialization

For numerical computation, the (ζ, η) space is discretized on a square grid of size *N* × *N* with sampling interval Δ,
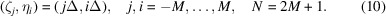
The discretized wavelength–angle distribution to be reconstructed is denoted by 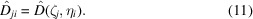
The grid spacing Δ is chosen to resolve the narrowest structure presented in the measured projections, while the total extent *N*Δ is selected to fully contain all nonzero projections. A hat symbol indicates an estimated quantity.

Because baseline-corrected and smoothed projections may still contain small negative values due to detector noise, a small positive constant ε > 0 is added to enforce non-negativity,
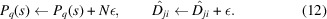
After convergence, ε is subtracted from the reconstructed distribution. The initial estimate 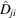 is set to a uniform constant value, introducing no prior bias.

#### 2.4.3. Rotation of the current estimate

For each projection angle *q*, a rotation operator *R*_*q*_ is defined to rotate the current estimate 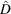 by an angle *q* in the counterclockwise direction on the discrete grid.

Standard image-rotation procedures leave undefined pixels near the corners of the rotated image. To maintain non-negativity and consistency of the grid, all undefined pixels are filled with the regularization constant ε. As a result, the rotated distribution 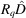 remains strictly positive and defined on the same grid.

#### 2.4.4. Forward projection, shift correction, and backprojection

The forward projection for a given angle *q* is calculated as the line sum of the rotated distribution, 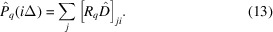
Owing to uncertainties in the effective projection angle, the measured projection *P*_*q*_(*s*), defined as a continuous function of *s*, may exhibit a lateral shift relative to the forward projection. The measured projection is therefore shifted to maximize its correlation with 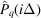 and then discretized onto the same grid, yielding *P*_*q*_(*i*Δ).

A multiplicative correction factor is defined as 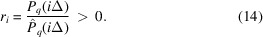
This factor is applied to the rotated distribution, 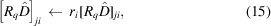
enforcing consistency between the updated projection and the measured data. The corrected distribution is then rotated back using *R*_−*q*_, completing a single update step.

#### 2.4.5. One OS-EM cycle

**Figure 4:**
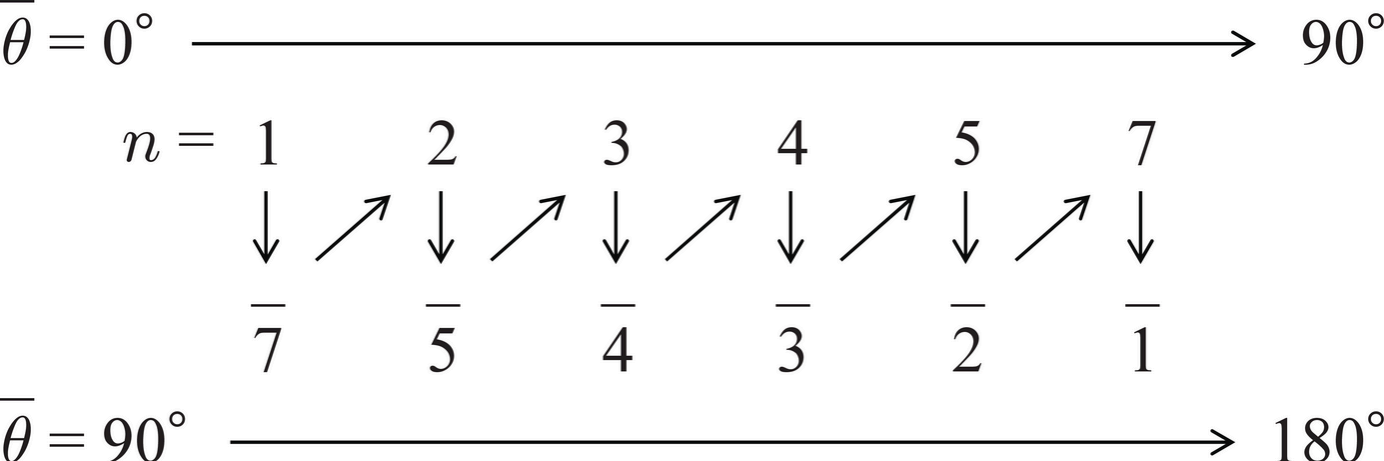
Ordering of projection angles within a single OS-EM cycle. Reflection indices *n* are grouped into upward- and downward-reflection geometries and arranged according to their Bragg angles from 0° to 180°. Reflections with *n* = ±6 were not observed. Projections are applied sequentially in an alternating manner to reduce directional bias. This example is used for the analysis for the beams from the lapped-crystal DCMs; for the beam from the polished-crystal DCM, the *n* = 1 projection was omitted because of excessive broadening.

A single OS-EM cycle consists of applying the above update sequentially to all available projection angles *q*. To reduce directional bias, the projection angles are ordered such that successive angles are as close to orthogonal as possible. When the aspect factor *a* is chosen to mitigate the limited-angle problem, this ordering can be defined almost uniquely, as illustrated in Fig. 4 ▸.

To reduce computational cost, the final inverse rotation *R*_−*q*_ in one update and the initial rotation 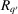 in the subsequent update are combined into a single rotation 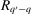.

As an exception, projection-shift corrections are omitted for the first two updates in the first OS-EM cycle. At this stage, the initially uniform distribution produces very broad forward projections, which are unsuitable for correlation-based shift estimation.

The OS-EM cycle is repeated until convergence of 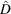 is achieved. After convergence, the regularization constant ε is subtracted from all pixels to obtain the final reconstructed wavelength–angle distribution.

## 3. Experimental setup

The experiments were performed using the optical configuration shown in Fig. 1 ▸, at beamline BL05XU of SPring-8. The beamline (Yumoto *et al.*, 2020 ▸) was formerly equipped with an in-vacuum planar undulator comprising 93 magnet pairs with a period of 32 mm, which produced the maximum intensity of 100 keV radiation at the 22nd harmonic for a magnet gap of 8.42 mm. The beam size was defined by an incident slit with openings of 0.56 mm (vertical) and 1.86 mm (horizontal), located 29 m downstream from the undulator. The beam impinged on the first crystal of the DCM at a distance of 46 m from the undulator, and the upwardly reflected beam was received by the second crystal positioned further 68 cm downstream. The monochromated beam delivered through a vacuum window was the one to be characterized.

The diffraction plane of the DCM was set to the lowest-index Si(111) reflection in order to maximize the beam intensity. The pair crystals in the DCM were selected from the mechanochemically polished crystals as a reference, and from additionally lapped crystals from mechanochemically polished condition. The grit numbers of lapping abrasive powders were #2000 (finest), #1200, #800, and #400 (coarsest). Each pair crystals were finished using the same abrasive grit. The process was performed by hand in a circular motion while maintaining a constant crystal orientation, until a uniform finish was obtained. Although the lapping was not controlled on the time-controlled basis, the net lapping time (excluding inspection time) was approximately half an hour per crystal. Because the etching of lapped surfaces cannot be controlled with sufficient precision, no additional etching was performed after the lapping process.

At 65 m from the undulator, an Si(111) analyzer crystal finished by mechanochemical polishing was mounted on the θ stage of a high-precision θ–2θ goniometer [RA20-21-1V, Kohzu Precision Co. Ltd; Fig. 1 ▸(*b*)]. Reflected beams were detected using an Si PIN photodiode (S14537-320, Hamamatsu Photonics KK, packaged by Ohyo Koken Kogyo Co. Ltd) mounted on the 2θ arm. Two slits with openings of 5 mm (vertical) and 10 mm (horizontal) were installed in front of the detector to suppress stray scatter.

The θ stage was driven by a stepper motor with a mechanical angular resolution of 1/50000° in a ten-subdivision mode. The 2θ arm was synchronized with the θ rotation. The θ stage was equipped with an angular encoder with a resolution of 1/40000°. Although this encoder resolution was slightly inferior to the driving step size, its linearity was more reliable.

Photodiode currents were converted to voltages using an amplifier. Because the peak intensities varied widely among different reflections, the amplifier gain was adjusted individually for each rocking-curve measurement. As a result, different baseline offsets and electronic noise levels were introduced, as discussed in §2.3.

Rocking-curve measurements using the analyzer crystal were performed in a continuous scan mode to reduce the total measurement time. Encoder counts were recorded at a sampling rate of 1 kHz, together with photodiode voltages averaged over 1 ms. Amplifier gains were changed at pre­determined timings during the scans. The θ rotation speeds were set to 0.006° s^−1^ for the beam from the polished-crystal DCM and 0.02° s^−1^ for the beams from the lapped crystal DCMs. Scans were performed over θ ranges 0–15° and 165–180°. The intermediate angular range, in which no visible reflection peaks were observed, was traversed at a higher speed. A full rotation from 0° to 180° took 35 min for the beams from the lapped-crystal DCMs.

## 4. Data preprocessing and determination of analysis parameters

### 4.1. For beams from lapped-crystal DCMs

Because the wavelength–angle distributions of the beam from the lapped-crystal DCMs were analyzed using the same procedure, the #1200 case is described here as a representative example. Although the numerical values of the analysis parameters—namely the sampling interval Δ, grid size *N*, and regularization constant ε—varied depending on the degree of wavelength–angle broadening, the procedure used to determine these parameters was common to all cases.

**Figure 5:**
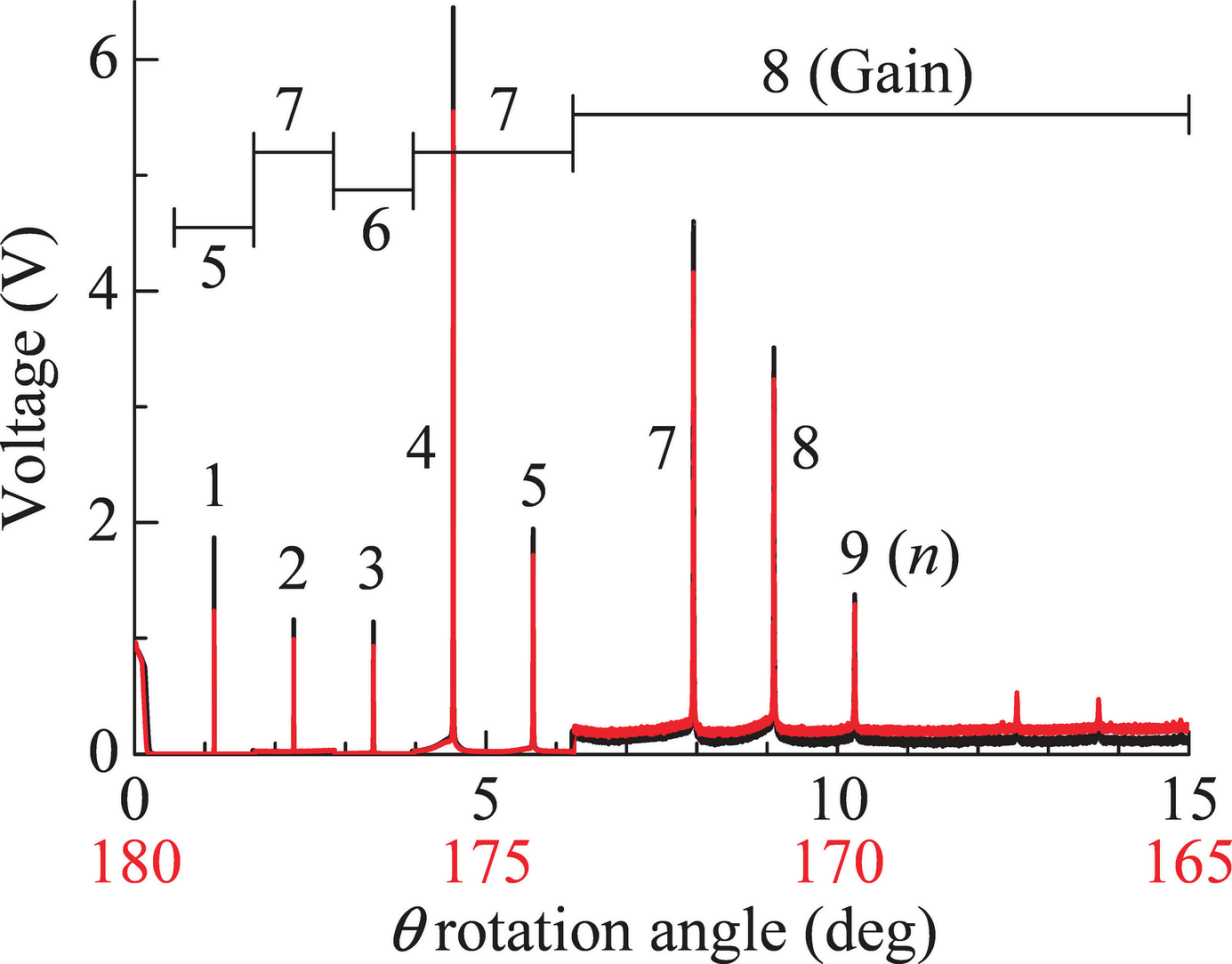
Intensity profile obtained from a full-rotation θ–2θ scan for the #1200 beam. The black curve corresponds to the θ range from 0° to 15°, and the red curve to the range from 165° to 180° (downward-reflection geometry), plotted in descending order of θ. The integers shown near the profile indicate the reflection order *n* of the *nnn* and 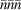 reflections, while those shown above indicate the exponent *m* of the amplifier gain (10^*m*^ V A^−1^). Peak positions were used to determine the mean wavelength of the beam.

Fig. 5 ▸ shows the intensity profile obtained from a full-rotation θ–2θ scan. The black curve corresponds to the θ range from 0° to 15°, while the red curve represents the range from 165° to 180° (downward-reflection geometry), plotted in descending order of θ. The integers shown near the profile indicate the reflection order *n* corresponding to the *nnn* and the 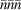 reflections, whereas those shown above the profile indicate the exponent *m* of the amplifier gain 10^*m*^ V A^−1^. The mean wavelength was calculated to be 0.12392 Å (photon energy 100.05 keV) from the peak positions.

**Figure 6:**
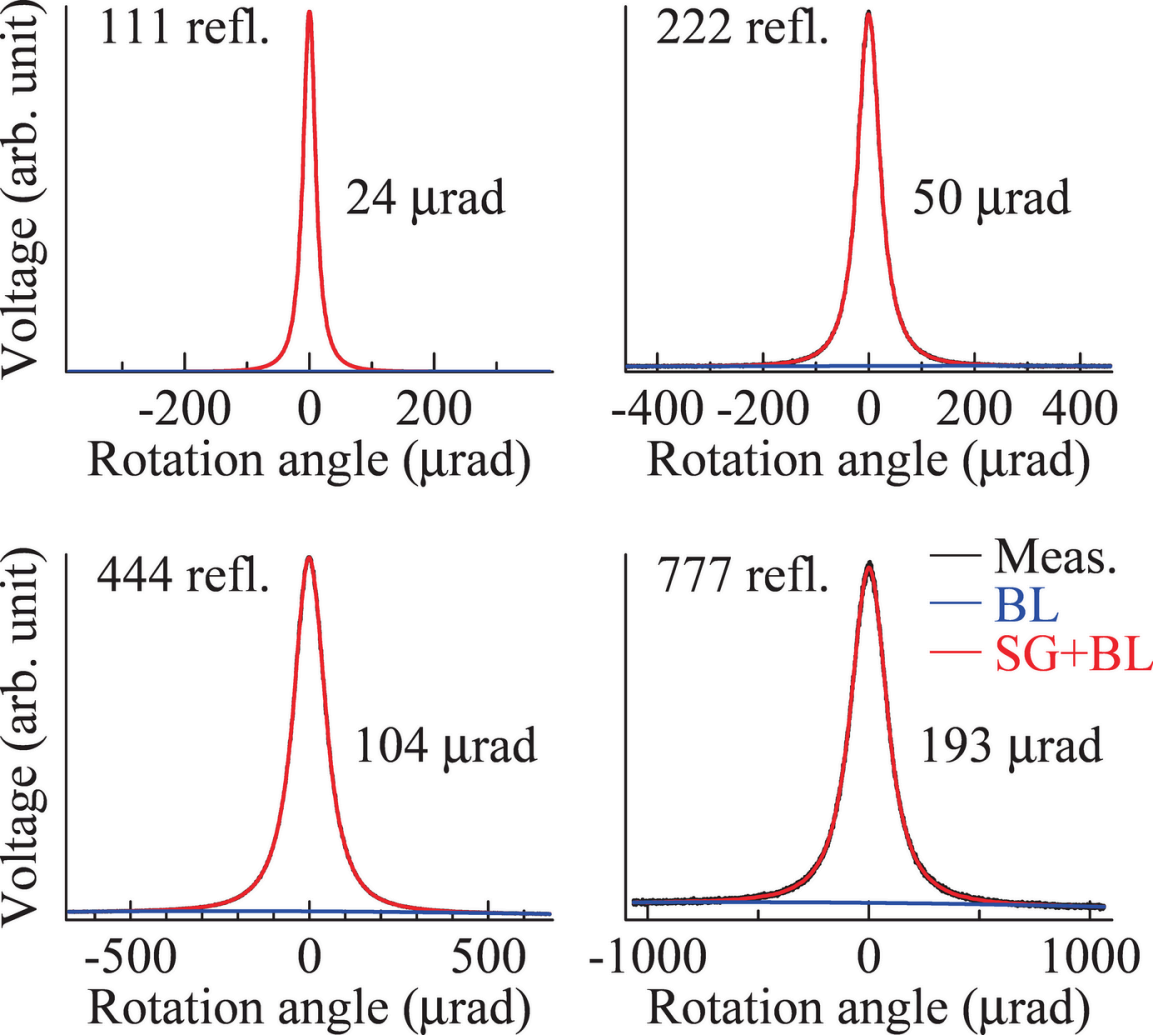
Magnified rocking-curve profiles for the *n* = 1, 2, 4 and 7 upward reflections for the #1200 beam, with each peak position aligned to zero. Measured data are shown by black curves, and the estimated baselines by blue curves. After baseline subtraction, the profiles were smoothed using second-order Savitzky–Golay filters. The red curves show the sums of the smoothed profiles and the baselines, illustrating that electronic noise was effectively suppressed without significant distortion of the peak shapes. The FWHMs of the smoothed profiles are indicated.

Fig. 6 ▸ shows magnified views of the rocking curves for the *n* = 1, 2, 4, and 7 upward reflections, with each peak position aligned to zero. The measured data are shown by black curves, and the estimated baselines by blue curves. After baseline subtraction, the data were smoothed using second-order Savitzky–Golay filters. The filter window lengths were chosen as odd integers corresponding to 0.2 times the FWHM of each rocking curve. The red curves show the sums of the smoothed profiles and the baselines, demonstrating that the smoothing effectively suppressed electronic noise without distorting the peak shapes.

The FWHM of the 111 reflection was 24 µrad, which is approximately ten times larger than its Darwin width of 2.5 µrad. The broadening by the analyzer crystal was therefore negligible, and explicit deconvolution was unnecessary. The 222 reflection is formally forbidden under the assumption of spherical electron density around each atom; however, deviations from this symmetry result in a finite reflected intensity. In the present measurements, the 222 reflection was weaker than the 555 reflection but stronger than the 777 reflection. For the 444 reflection, although a broad structure appears in Fig. 5 ▸, the magnified view reveals a distinct peak superimposed on a flat background. The 777 reflection exhibited a sufficiently high signal-to-noise ratio for reliable use in the reconstruction.

Using the reconstruction conditions defined in §2.4.2, the analysis parameters were chosen as Δ = 2.19 × 10^−5^, *N* = 655, and ε = 1 × 10^−7^. The reconstructed region covered relative wavelength deviations of |ζ| ≤ 7.2 × 10^−3^ (±0.72%) and angular deviations of |ξ| ≤ 360 µrad before applying the aspect factor. The OS-EM cycle was repeated 1000 times to ensure full convergence.

### 4.2. For beam from polished-crystal DCM

For this beam, the 111 rocking curve of the analyzer crystal was excluded from the reconstruction dataset because its width was comparable with the Darwin width, making deconvolution ill-posed. The window lengths of the second-order Savitzky–Golay filters were set to 0.1 times the FWHMs of the corresponding rocking curves. Because the wavelength–angle distribution was much sharper than those from the lapped-crystal DCMs, some rocking curves exhibited Gibbs-type oscillations when larger window lengths were used. The reduced window length effectively suppressed these artifacts while preserving the intrinsic peak shapes.

## 5. Results

**Figure 7:**
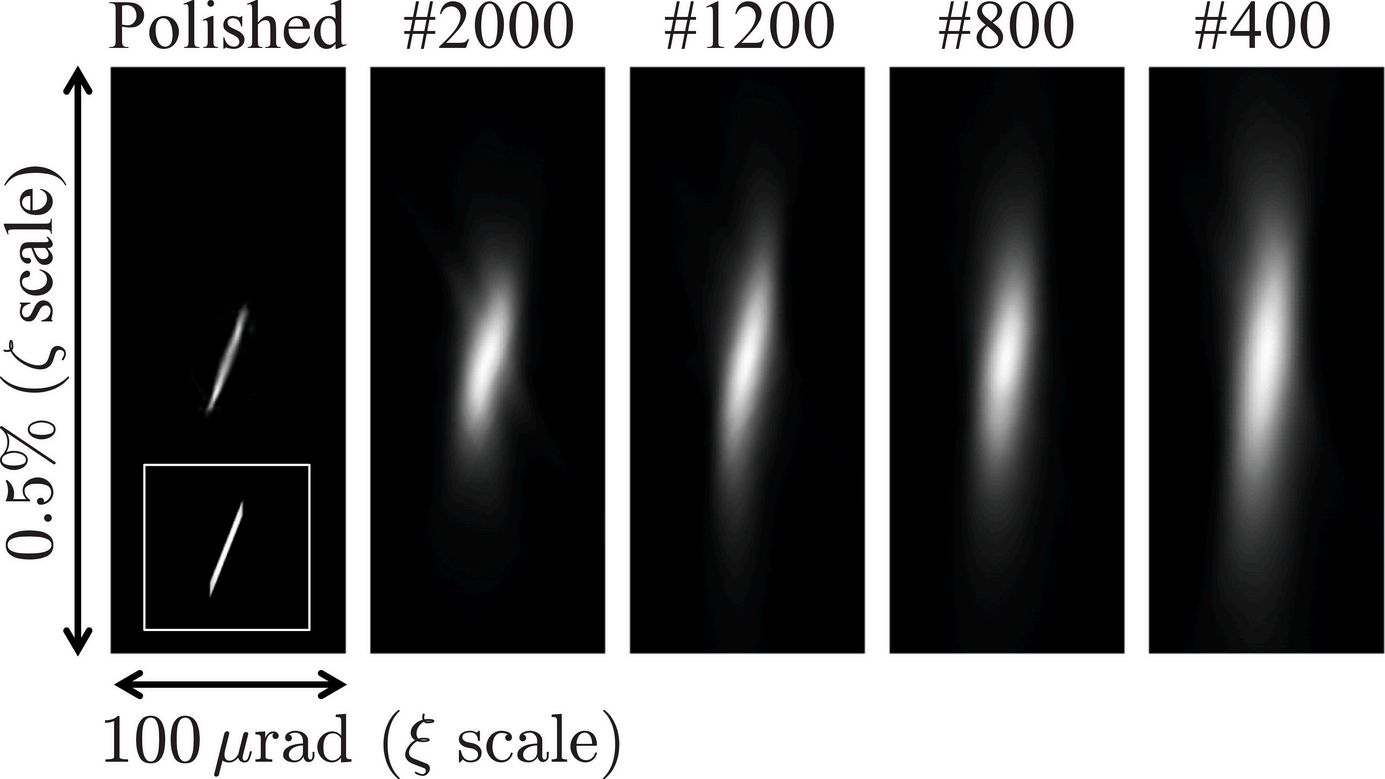
Reconstructed wavelength–angle distributions for the beams produced using the polished- and lapped-crystal DCMs. The vertical axis represents the relative wavelength deviation ζ, and the horizontal axis represents the angular deviation. The displayed ranges are 0.5% in wavelength and 100 µrad in angle. The intensity scale is normalized independently for each distribution. For the polished-crystal case, the distribution calculated using the DuMond analysis is shown below the reconstructed distribution and enclosed by a white frame.

Fig. 7 ▸ shows the reconstructed wavelength–angle distributions 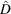 for beams produced using the polished- and lapped-crystal DCMs. In each panel, the intensity scale is normalized independently. The vertical axis represents the relative wavelength deviation ζ, with zero at the center and positive values upward, while the horizontal axis represents the angular deviation. The displayed ranges correspond to 0.5% in wavelength and 100 µrad in angle.

The leftmost panel in Fig. 7 ▸ shows the reconstructed distribution of the beam from the polished-crystal DCM. The distribution calculated using the DuMond analysis is shown below it and enclosed by the white frame. In the calculation, the source properties were taken from the synchrotron radiation calculator *SPECTRA*, Version 12 (Tanaka & Kitamura, 2001 ▸; Tanaka, 2021 ▸). The reconstructed distribution agrees well with the calculated one, reproducing the wavelength–angle coupling imposed by Bragg’s law.

The reconstructed distribution of the beam from the polished-crystal DCM exhibits a narrow angular extent. The angular divergence, estimated from the projection of the distribution, was 13 µrad (FWHM), while the value obtained from the DuMond calculation was 14 µrad. Both values are smaller than the angular acceptance defined by the incident slit, 0.56 mm/29 m ≃ 19 µrad. This indicates that the angular spread of the beam was limited by the source properties. Minor differences between the reconstructed and calculated distributions are attributed to uncertainties in the simulation parameters or to instabilities in the DCM during operation.

For the beams from the lapped-crystal DCMs, the reconstructed wavelength–angle distributions exhibit systematic changes with surface finishing, as shown in Fig. 7 ▸. Compared with the polished-crystal case, the distributions become increasingly elongated along the wavelength direction as the grit number decreases. In contrast, the angular extent remains similar among the lapped-crystal DCMs. This behavior suggests that the double-crystal geometry effectively constrains the outgoing angular divergence.

The wavelength spreads and angular divergences were quantified by taking line integrals of the reconstructed distributions along the angular and wavelength directions, respectively. Table 1 ▸ summarizes the FWHMs of the wavelength spreads and angular divergences, together with the incident beam intensities measured independently.

In the #2000 case, the wavelength–angle coupling characteristic of the polished-crystal DCM is largely preserved, although the distribution is broadened. The wavelength spread and angular divergence increased by factors of 1.5 and 1.6, respectively, relative to the polished-crystal case, while the beam intensity increased by a factor of 7. As the grit number decreases further, the wavelength spread increases monotonically, whereas the angular divergence remains approximately constant at around 20 µrad.

**Figure 8:**
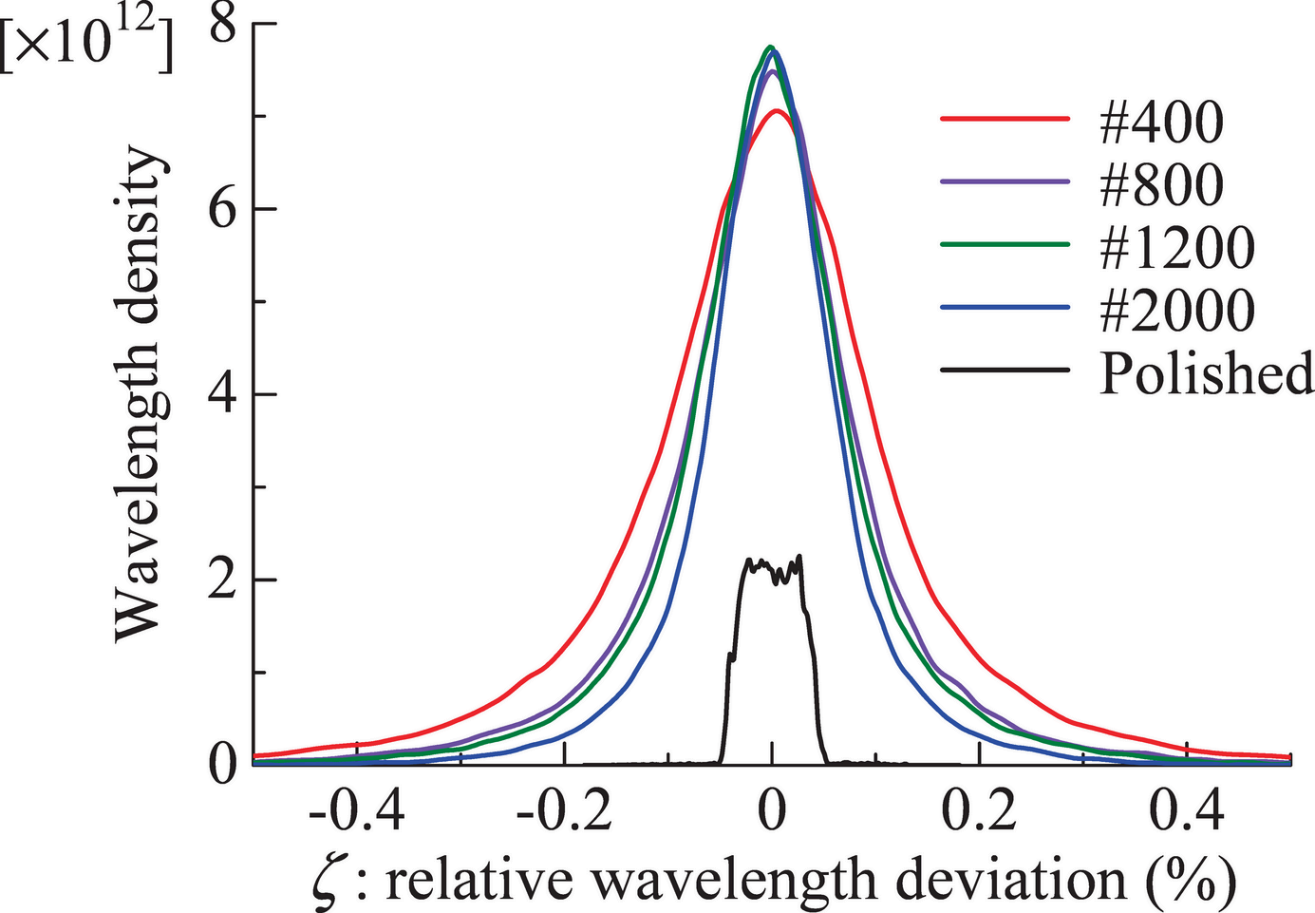
Wavelength distributions obtained by integrating the reconstructed wavelength–angle distributions over the angular direction and normalizing by the corresponding incident beam intensities. The horizontal axis represents the relative wavelength deviation ζ, and the vertical axis represents the photon flux density per wavelength bin of Δζ = 0.1%.

Fig. 8 ▸ shows the wavelength distributions normalized by the corresponding incident beam intensities. The wavelength spread increases systematically with decreasing grit number.

## 6. Discussion

### 6.1. Applicability of wavelength–angle characterization

The wavelength–angle characterization developed in this study combines full-rotation rocking-curve measurements with an iterative tomographic reconstruction scheme. Although the available projections are discrete and sparse owing to crystallographic constraints of the analyzer crystal, the present results demonstrate that physically meaningful wavelength–angle distributions can be reconstructed for high-energy X-ray beams under appropriate experimental conditions.

A key validation of the method is provided via the characterization of the beam from the polished Si(111) DCM. For this beam, the reconstructed wavelength–angle distribution agrees well with that calculated using the DuMond analysis, which is only applicable to the polished-crystal DCM. Although the method is formulated on the basis of the DuMond analysis, this validation is not circular. The DuMond analyses are independently applied to the DCM crystals and the analyzer crystal, and therefore there is no causal relationship. The agreement indicates that the reconstruction retains sufficient redundancy despite the limited number of projections and supports the reliability of the present approach when applied to beams with intrinsically simple wavelength–angle distributions, as typically required for general-purpose X-ray probes.

The present analysis is intended for beams whose rocking curves are well separated. Beams that do not meet this condition exhibit excessively broad wavelength spreads or angular divergences, which thus complicate baseline correction and degrade the reliability of the reconstructed projections. However, the present analysis would be unnecessarily sophisticated for such beams, for which a rougher characterization would generally be sufficient.

For high-energy X-ray beams, the limited angular coverage associated with small Bragg angles is an inherent experimental constraint. In the present study, this limitation was mitigated by introducing an aspect factor to rescale the wavelength–angle space, thereby improving the practical distribution of projection angles. This scaling substantially broadens the range of beam conditions under which stable reconstructions can be achieved.

An important practical advantage of the present approach is that it does not require explicit deconvolution of the intrinsic reflection profile of the analyzer crystal. Provided that the measured rocking-curve widths significantly exceed the Darwin width, the intrinsic broadening can be neglected, and data that would otherwise require ill-posed deconvolution can simply be excluded from the reconstruction. This strategy enhances the robustness of the method and facilitates its application to experimental data obtained under realistic beamline conditions.

Within this defined scope, the present wavelength–angle analysis provides a practical and reliable diagnostic tool for evaluating high-energy X-ray beams whose properties cannot be predicted quantitatively by existing theoretical models. In particular, this analysis provides a quantitative framework for assessing how surface processing of monochromator crystals influences beam properties, and it offers a basis for optimizing lapping conditions for specific experimental requirements.

### 6.2. Key issues toward realistic modeling for lapped-crystal DCMs

The reconstructed wavelength–angle distributions shown in Fig. 7 ▸ provide experimental constraints that any realistic model for lapped Si crystal DCMs must satisfy. In particular, the systematic evolution of the distributions with surface roughening indicates that lapped crystals should be regarded as intermediate states between perfect crystals and fully mosaic crystals, rather than being described adequately by either limiting case alone.

X-ray diffraction in crystals arises from the interference of elastically scattered waves, such that incident rays satisfying the Bragg condition are reflected while others are transmitted. An increase in wavelength spread therefore implies an expansion of the effective wavelength acceptance of the monochromator. According to Bragg’s law, such broadening can originate from variations in lattice spacing and crystal orientation, which are commonly associated with increased mosaicity. This interpretation is consistent with earlier reports by Shiwaku *et al.* (1991 ▸, 1992 ▸), which showed that coarser lapping leads to higher integrated reflectivity in single-reflection configurations.

In a double-crystal geometry, however, the second crystal acts as an additional wavelength–angle filter. Rays transmitted through both crystals must satisfy the Bragg conditions of the reflecting domains in each crystal, which imposes constraints on the transmitted wavelength–angle distribution in DuMond space. The reconstructed distributions indicate that, despite the systematic increase in wavelength spread with surface roughening, the angular divergence of the exiting beam remains nearly unchanged. This observation implies that the double-crystal geometry constrains the outgoing angular acceptance even when the individual crystals exhibit significant surface damage.

The transmission through two successive crystals requires not only angular matching between reflecting domains but also spatial overlap along the beam path. Increased mosaicity may reduce the probability of such double matching. Nevertheless, the present results show that the net beam intensity continues to increase with surface roughening, indicating that additional factors contribute to the observed gain. One possible factor may involve the high transmissivity of high-energy X-rays. Electron microscopy studies of lapped Si surfaces (Wu *et al.*, 1992 ▸) have revealed complex damaged-layer structures, including cracking, local amorphization, fragmentation into small crystalline blocks, and misorientation. High-energy X-rays are largely transparent to amorphous or highly distorted regions, which may allow rays reflected by domains in the first crystal to propagate through damaged layers of the second crystal until encountering suitably oriented domains.

The identification of the mechanisms responsible for these constraints remains an open problem. Nevertheless, the wavelength–angle distributions obtained in this study provide a quantitative experimental basis for future theoretical and experimental investigations aimed at developing realistic models of lapped-crystal DCMs and optimizing surface-processing conditions for specific experimental requirements.

## 7. Conclusion

A method for experimentally determining the wavelength–angle distributions of high-energy X-ray beams has been developed by combining full-rotation θ–2θ rocking-curve measurements with an iterative tomographic reconstruction procedure. In this framework, each rocking curve is interpreted as a projection of the wavelength–angle distribution in DuMond space, enabling reconstruction from a discrete set of crystallographically determined projection angles.

The method was applied to 100 keV X-ray beams produced using both a conventional surface-polished Si(111) DCM and lapped Si(111) crystals with various degrees of surface roughness. For the beam from polished-crystal DCM, the reconstructed wavelength–angle distribution agrees well with that predicted by the DuMond analysis, validating the reconstruction approach. For the beams from the lapped-crystal DCMs, the reconstructed wavelength–angle distributions reveal systematic modifications of beam properties associated with surface roughening. In particular, lapping the Si(111) crystals in a DCM systematically increases the wavelength spread of the transmitted beam, while the angular divergence remains nearly unchanged under the double-crystal geometry.

The wavelength–angle characterization presented here provides a practical and robust diagnostic framework for evaluating high-energy X-ray beams under realistic beamline conditions. By reconstructing beam properties in DuMond space, the present study provides quantitative experimental constraints on the wavelength–angle distributions of beams produced by lapped-crystal DCMs. These constraints form a basis for future theoretical and experimental investigations aimed at developing realistic models of lapped crystals and optimizing surface-processing conditions to meet specific experimental requirements.

## Figures and Tables

**Table 1:** Incident beam intensities, wavelength spreads, and angular divergences for the beams produced using the polished- and the lapped-crystal DCMs The wavelength spreads and angular divergences are given as FWHMs derived from the reconstructed wavelength–angle distributions.

Item	Polished	#2000	#1200	#800	#400
Intensity (×10^13^ photons s^−1^)	0.17	1.2	1.4	1.5	1.8
Wavelength spread (%)	0.08	0.12	0.14	0.16	0.21
Angular divergence (µrad)	13	21	20	21	24

## Data Availability

The data supporting this study are available from the corresponding author upon reasonable request.
